# Association of electronic cigarette use and suicidal behaviors: a systematic review and meta-analysis

**DOI:** 10.1186/s12888-024-06012-7

**Published:** 2024-09-10

**Authors:** Abdelaziz A. Awad, Ramaiah Itumalla, Abhay M. Gaidhane, Mahalaqua Nazli Khatib, Suhas Ballal, Pooja Bansal, Manish Srivastava, Isha Arora, MRavi Kumar, Aashna Sinha, Kumud Pant, Hashem Abu Serhan, Muhammed Shabil

**Affiliations:** 1 Faculty of Medicine, Al-Azhar University, Cairo, 11651 Egypt; 2 School of Management, The Apollo University, Chittoor, Andhra Pradesh 517127 India; 3 School of Epidemiology and Public Health, Jawaharlal Nehru Medical College, and Global Health Academy, Datta Meghe Institute of Higher Education, Wardha, India; 4 Division of Evidence Synthesis, Global Consortium of Public Health and Research, Datta Meghe Institute of Higher Education, Wardha, India; 5 Department of Chemistry and Biochemistry, School of Sciences, JAIN (Deemed to Be University), Bangalore, Karnataka India; 6 Department of Allied Healthcare and Sciences, Vivekananda Global University, Jaipur, Rajasthan 303012 India; 7 Department of Cardiology, NIMS University, Jaipur, India; 8 Chandigarh Pharmacy College, Chandigarh Group of College, Jhanjeri, Mohali, Punjab 140307 India; 9 Department of Chemistry, Raghu Engineering College, Visakhapatnam, Andhra Pradesh 531162 India; 10 School of Applied and Life Sciences, Division of Research and Innovation, Uttaranchal University, Dehradun, India; 11 Department of Biotechnology, Graphic Era (Deemed to Be University), Clement Town Dehradun, 248002 India; 12 Department of Allied Sciences, Graphic Era Hill University Clement Town Dehradun, Clement Town Dehradun, 248002 India; 13 Department of Ophthalmology, Hamad Medical Corporation, Doha, Qatar; 14 Center for Global Health Research, Saveetha Medical College and Hospital, Saveetha Institute of Medical and Technical Sciences, Saveetha University, Chennai, India; 15 Medical Laboratories Techniques Department, AL-Mustaqbal University, Hillah, Babil 51001 Iraq

**Keywords:** Electronic cigarettes, Mental health, Adolescent health, Suicide, Self-harm, Nicotine, Public health, Good health and well-being, Global health targets, Suicide prevention

## Abstract

**Background:**

The proliferation of electronic cigarettes (e-cigarettes) has presented new challenges in public health, particularly among adolescents and young adults. While marketed as safer than tobacco and as cessation aids, e-cigarettes have raised concerns about their long-term health and psychosocial impacts, including potential links to increased suicidal behaviors. This study aims to evaluate the relationship between e-cigarette use and suicidal behaviors by conducting a systematic review of the current literature.

**Methods:**

We searched PubMed, Web of Science, and EMBASE for studies up to March 10, 2024, examining the relationship between e-cigarette use and suicidal behaviors. Eligible studies included cross-sectional, longitudinal, retrospective, prospective, and case–control designs. Meta-analysis was performed to calculate pooled odds ratios (ORs). Newcastle Ottawa scale was used to assess the quality of studies. R software (V 4.3) was used to perform the meta-analysis.

**Results:**

Our analysis included fourteen studies, predominantly from the US and Korea, with participants ranging from 1,151 to 255,887. The meta-analysis identified a significant association between e-cigarette use and an increased risk of suicidal ideation (OR = 1.489, 95% CI: 1.357 to 1.621), suicide attempts (OR = 2.497, 95% CI: 1.999 to 3.996), and suicidal planning (OR = 2.310, 95% CI: 1.810 to 2.810). Heterogeneity was noted among the studies.

**Conclusion:**

E-cigarette use is significantly associated with the risk of suicidal behaviors, particularly among adolescents. The findings underscore the necessity for caution in endorsing e-cigarettes as a safer smoking alternative and call for more extensive research to understand the underlying mechanisms. Public health strategies should be developed to address and mitigate the risks of suicidal behaviors among e-cigarette users.

**Supplementary Information:**

The online version contains supplementary material available at 10.1186/s12888-024-06012-7.

## Introduction

The advent of electronic cigarettes (e-cigarettes) has ushered in a new era in the landscape of tobacco consumption, offering a technological alternative to traditional smoking methods [1–4]. Marketed as a safer option and a potential tool for smoking cessation, e-cigarettes have rapidly gained popularity among various demographics, notably among teens and young adults [5]. However, this rise in e-cigarette use has been paralleled by growing concerns among health professionals and researchers regarding their long-term health implications and psychosocial outcomes [6–11].

The use of e-cigarettes, especially among adolescents and young adults, poses significant health concerns [12]. While e-cigarettes are often marketed as a safer alternative to traditional tobacco products, they are not without risks [11, 13]. Studies have shown that e-cigarette use can lead to increased exposure to harmful chemicals such as nicotine, which can adversely affect brain development in youths and lead to addiction. Additionally, there is evidence linking e-cigarette use with respiratory issues and the potential for cardiovascular harm [14]. The prevalence of e-cigarette use among adolescents and young adults has been rising, driven by factors such as appealing flavors, targeted marketing, and the perception of reduced harm compared to smoking [15]. This trend is alarming, as early exposure to nicotine can establish long-lasting addictive behaviors, potentially leading to the use of other tobacco products and sustaining a cycle of dependence that can be difficult to break.

Among the most alarming potential consequences is the impact of e-cigarette use on mental health, a subject that has recently begun to garner attention within the scientific community [16, 17]. Suicidal behaviors, encompassing suicidal ideation, planning, attempts, and completed suicide, represent a significant public health issue worldwide [18]. The World Health Organization (WHO) reports that nearly 700,000 people pass away from suicide each year, positioning it as the second most common cause of death among individuals aged 15 to 29 years. [19]. The etiology of suicidal behaviors is multifaceted, with a complex interplay of psychological, genetic, and environmental and social factors [20, 21]. Recently, substance use, including the consumption of nicotine through smoking, has been identified as a risk factor for suicidal behaviors [22–24]. Nicotine's neurobiological effects, which can influence mood and cognitive function, alongside the psychosocial aspects of substance use, may contribute to this association [23, 25].

The emergence of e-cigarettes, which deliver nicotine without the combustion of tobacco, was initially met with optimism for their potential to reduce the harm associated with traditional cigarette smoking [26, 27]. Nonetheless, the impact of e-cigarettes on mental health, and specifically their association with suicidal behaviors, remains underexplored and poorly understood. Preliminary studies have suggested a potential association between e-cigarette use and increased risk of suicidal behaviors [28–31]. Some studies have found that e-cigarette use is associated with increased suicidal ideation, suicide planning, and suicide attempts among adolescents. For instance, a study in South Korea found that among male participants, rates of suicidal ideation, suicide planning, and suicide attempts were higher among those who initially used e-cigarettes compared to those who initially used conventional cigarettes or never smoked [32]. Similarly, a scoping review of vaping and mental health found that e-cigarette use was associated with depression, suicidal ideation, and suicide attempts among adolescents [33]. Additionally, a study in the US found that the use of e-cigarettes was associated with 23% increased odds of seriously considering attempting suicide in the prior year among more than 25,000 adolescents participating in the US Youth Risk Behavior Survey [34]. These findings suggest that e-cigarette use may be a significant risk factor for suicide behaviors globally.

A systematic review has yet to be conducted to assess this issue. This systematic review and meta-analysis aimed to address this gap by evaluating the existing body of research on the association between e-cigarette use and suicidal behaviors. By aggregating data from diverse studies, this work seeks to provide a more robust understanding of the relationship. Through this analysis, we endeavor to contribute valuable insights into the potential psychosocial risks associated with e-cigarette use, offering a foundation for future research and informing policy-makers and healthcare providers in their efforts to mitigate the adverse outcomes related to e-cigarette consumption.

## Methods

This systematic review and meta-analysis adhered to the guidelines of the Preferred Reporting Items for Systematic Reviews and Meta-Analyses (PRISMA) to ensure transparency and completeness in reporting [35] (Table S1). The aim was to systematically review and synthesize existing literature on the association between e-cigarette use and suicidal behaviors, including suicidal ideation, planning, attempts, and completion. This systematic review has been registered prospectively in PROSPERO.

### Search strategy

A search strategy was developed to identify studies that investigated the association between e-cigarette use and suicidal behaviors. Electronic databases, including PubMed, Web of Science, and EMBASE, were searched from their inception to March 10, 2024. The search strategy combined terms related to e-cigarettes (e.g., "electronic cigarettes," "e-cigarettes," "vaping") with terms related to suicidal behaviors (e.g., "suicide," "suicidal" AND “self-harm"). Both MeSH terms (where applicable) and free text terms were used. The search strategy is displayed in Table S2.

### Eligibility criteria

For inclusion, we considered studies involving participants from the general population without any restrictions, thus allowing for a broad and inclusive analysis. The exposure of interest was clearly defined as the use of e-cigarettes, vaping products, or Electronic Nicotine Delivery Systems (ENDS), excluding studies that focused solely on traditional tobacco smoking due to its well-documented effects and distinct mechanisms compared to e-cigarettes. Regarding outcomes, we aimed to include studies that investigated suicidal ideation, suicidal planning, and suicidal attempts, explicitly excluding research that focused on non-suicidal self-harms to maintain a clear focus on suicidal behaviors directly. In terms of study design, our criteria included a wide range of observational studies, encompassing cross-sectional, longitudinal, retrospective, prospective, and case–control studies. We excluded qualitative studies, policy analyses, opinion pieces, case studies, case reports, reviews, and animal studies from our analysis, as these did not offer the empirical evidence necessary to address our research question directly (Table S3).

### Study selection

Titles and abstracts were screened for eligibility independently by two reviewers, utilizing Nested-Knowledge web software for the process. Subsequently, full texts of studies that appeared potentially suitable were retrieved and assessed independently for inclusion. Any discrepancies were settled through discussion or by consulting a third reviewer.

### Data extraction

Data extraction was conducted independently by two reviewers utilizing a uniform data extraction template. The information extracted encompassed characteristics of the study (such as author, publication year, country, design of the study, and size of the sample), demographics of the participants, and outcomes pertaining to suicidal behaviors. Discrepancies encountered during the data extraction process were addressed through discussions or by seeking the opinion of a third reviewer.

### Quality assessment

The assessment of the quality of the included studies was carried out using the Newcastle–Ottawa Scale. This scale assesses the selection criteria of the study groups, the comparability between these groups, and the determination of the exposure or outcome of interest.

### Statistical analysis

The meta-analysis employed a random-effects model (REM) to accommodate expected variability among the studies, offering a more standardized estimate of the overall effect. This approach is designed to manage the inherent differences found across studies. The relationship between the use of e-cigarettes and suicidal behaviors was measured using odds ratios (ORs) accompanied by 95% confidence intervals (CIs). The degree of heterogeneity between the studies was evaluated using the I^2^ statistic [36]. Additionally, a 95% prediction interval was utilized to provide deeper insight into the degree of heterogeneity. To examine publication bias, funnel plots and Egger's test were applied. The analysis was conducted using the ‘Meta’ and ‘Metafor’ packages within the R statistical software (Version 4.3) [37].

## Result

### Literature search

**Fig. 1:**
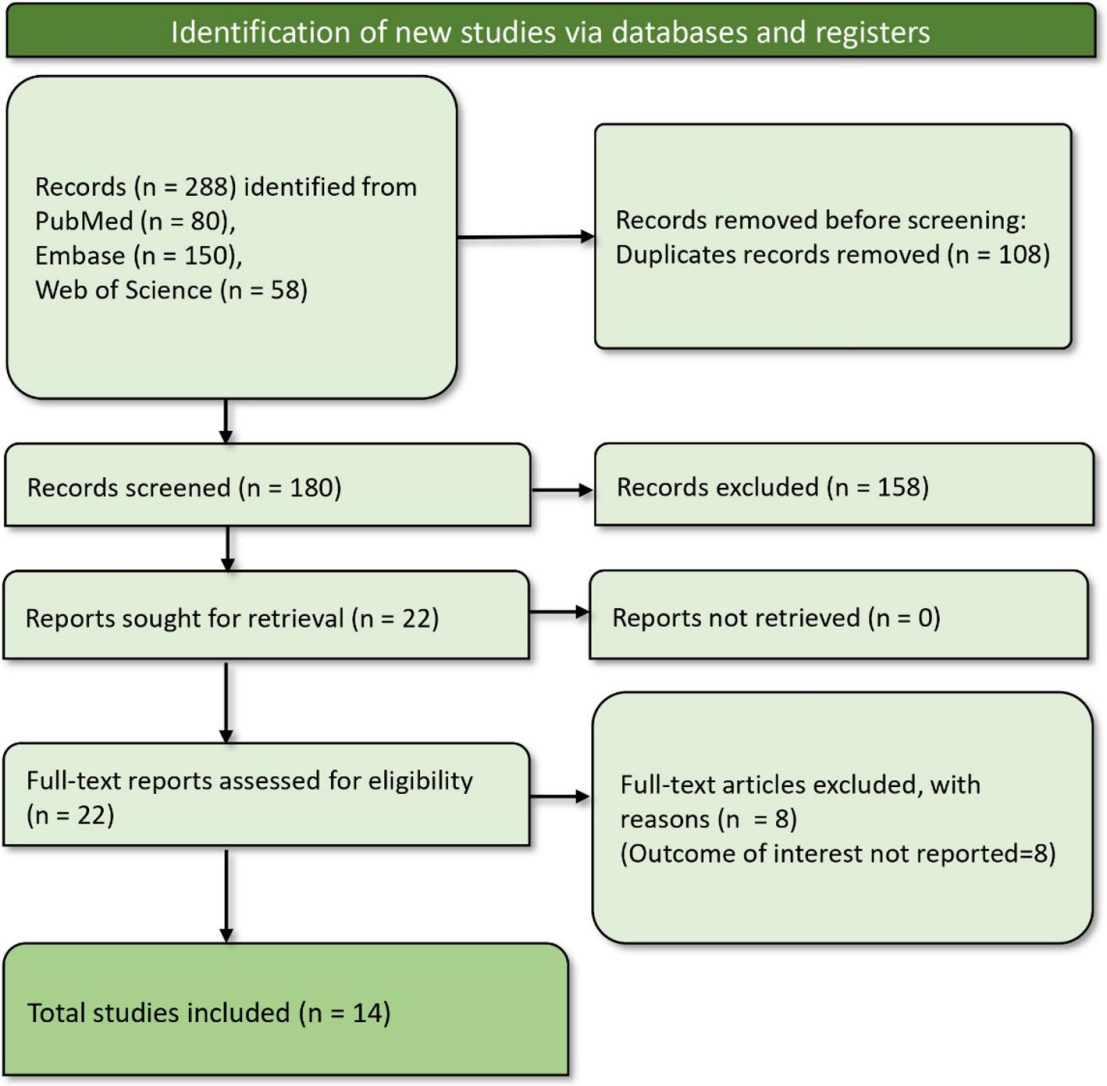
PRISMA flow diagram showing article screening and study selection process

The initial search yielded 288 records, which were reduced to 180 after the removal of 108 duplicate entries. Each of these records was screened based on our predefined criteria, identifying 22 potentially relevant reports for full retrieval. Upon closer examination, eight of these were excluded due to their lack of reporting on the outcomes of interest, specifically related to suicidal behaviors in the context of e-cigarette use. The screening process culminated in selecting 14 suitable studies [28–32, 38–46] for inclusion in our systematic review and meta-analysis (Fig. 1).

### Characteristics of included studies

The included studies in this systematic review primarily focus on the United States (US) and Korea, with a single study from Canada (Table 1). These studies employed a cross-sectional design to investigate the association between e-cigarette use and suicidal behaviors across diverse demographic groups, including active-duty service members, adolescents, bisexual high school students, asthmatic adolescents, the general population, and middle and high school students. The sample sizes vary significantly, ranging from 1,151 to 255,887 participants, with most studies reporting a balanced gender distribution where applicable.

**Table 1:** Characteristics of included studies

Study	Country	Design	Population	Sample size	Male%	OR (95% CI) for Suicide ideation	OR (95% CI) for Suicide plan	OR (95% CI) for Suicide attempt
Ahmed 2023 [38]	USA	Cross-sectional	Active-duty service members from the Army, Navy, Air Force, Marine Corps, and Coast Guard	17,166	83.31	2.00 (1.38–2.88)	NA	2.64 (1.14–6.08)
Baiden 2022 [28]	USA	Cross-sectional	High school students	14,285	49.7	1.55 (1.30–1.86)	1.62 (1.34–1.97)	1.75 (1.41–2.18)
Chadi 2019 [29]	USA	Cross-sectional	Adolescents	26,821	48.7	1.23 (1.03–1.47)	NA	NA
Dunn 2023 [39]	USA	Cross-sectional	Bisexual high school students	1151	NA	1.72 (1.20–2.48)	1.59 (1.11–2.28)	NA
Erhabor 2023 [40]	USA	Cross-sectional	Adolescents	12,767	50.50%	1.40 (1.13–1.74)	NA	NA
Huh 2021 [30]	Korea	Cross-sectional	Adolescents	57,069	52%	2.29 (1.44–3.65)	3.38 (1.81–6.33)	4.26 (2.16–8.38)
Jacobs 2021 [41]	USA	Cross-sectional	High School Students	12,578	48.2	NA	NA	1.43 (0.88–2.32)
Kim 2020 [31]	Korea	Cross-sectional	Asthmatic adolescents	195,847	NA	1.28 (1.18–1.43)	NA	2.11 (1.51–2.95)
Kim 2021 [32]	Korea	Cross-sectional	Adolescents	255,887	51.20%	Male = 1.46 (1.27–1.68), Female = 1.57 (1.26–1.95)	Male = 2.15 (1.79–2.57), female = 2.38 (1.82–3.12)	Male = 2.47 (1.96–3.11), Female = 3.09 (2.31–4.15)
Kim 2021 [43]	Korea	Cross-sectional	General population	39,225	NA	1.569 (1.839–3.588)	3.220 (2.001–5.181)	4.271 (2.041–8.939)
Kim 2021 [42]	Korea	Cross-sectional	Middle and high school students	5405	81.1	1.58 (1.31–1.89)	2.44 (1.94–3.08)	2.44 (1.85–3.22)
Lee 2019 [44]	Korea	Cross-sectional	Adolescents	62,276	50.7	Total = 2.49 (1.82–3.42), Male = 2.11 (1.45–3.06), Female = 4.05 (2.12–7.72)	Total = 4.63 (3.22–6.67), male = 3.08(1.94–4.91), female = 11.9 (6.28–22.55)	Total = 6.17 (4.13–9.24), Male = 3.64(2.07–6.39), female = 16.42 (8.5–31.71)
Pham 2020 [45]	Canada	Cross-sectional	General population	53,050	48.9	Female = 2.2(0.8–6.0), Male = 2.3(1.0–5.4)	NA	NA
Welty 2023 [46]	USA	Cross-sectional	High school students	10,520	NA	NA	NA	3.0 (2.1–3.9)

*Abbreviations*: *CI* Confidence Interval, *NA* Not Available, *OR* Odds Ratio

Ahmed's 2023 study [38] on active-duty service members from the US military branches reports an OR of 2.00 for suicide ideation and 2.64 for suicide attempts. Baiden's 2022 [28] research on US high school students shows ORs ranging from 1.55 for suicide ideation to 1.75 for suicide attempts, with explicit data on planning as well. Chadi's 2019 study [29], Dunn's 2023 study [39], and Erhabor's 2023 study [40], all based in the US, focus on adolescents and specific groups such as bisexual students, offering varying ORs for suicide ideation and plans. Huh's 2021 study [30] stands out with its Korean adolescent population, presenting higher ORs, especially for suicide attempts, peaking at 4.26. Kim's series of studies from 2020 and 2021 in Korea explore different populations, including asthmatic adolescents and the general population, with ORs for suicide ideation and attempts indicating a considerable risk associated with e-cigarette use. Lee's 2019 [44] study from Korea provides a detailed breakdown by gender, revealing a stark contrast in ORs for suicide attempts between males and females. Pham's 2020 study [45] is the only Canadian entry offering gender-specific ORs for suicide ideation. Finally, Welty's 2023 study [46] focuses on US high school students, providing data exclusively for suicide attempts with an OR of 3.0. The quality assessment of studies is given in Table S4.

### Suicide ideation

**Fig. 2:**
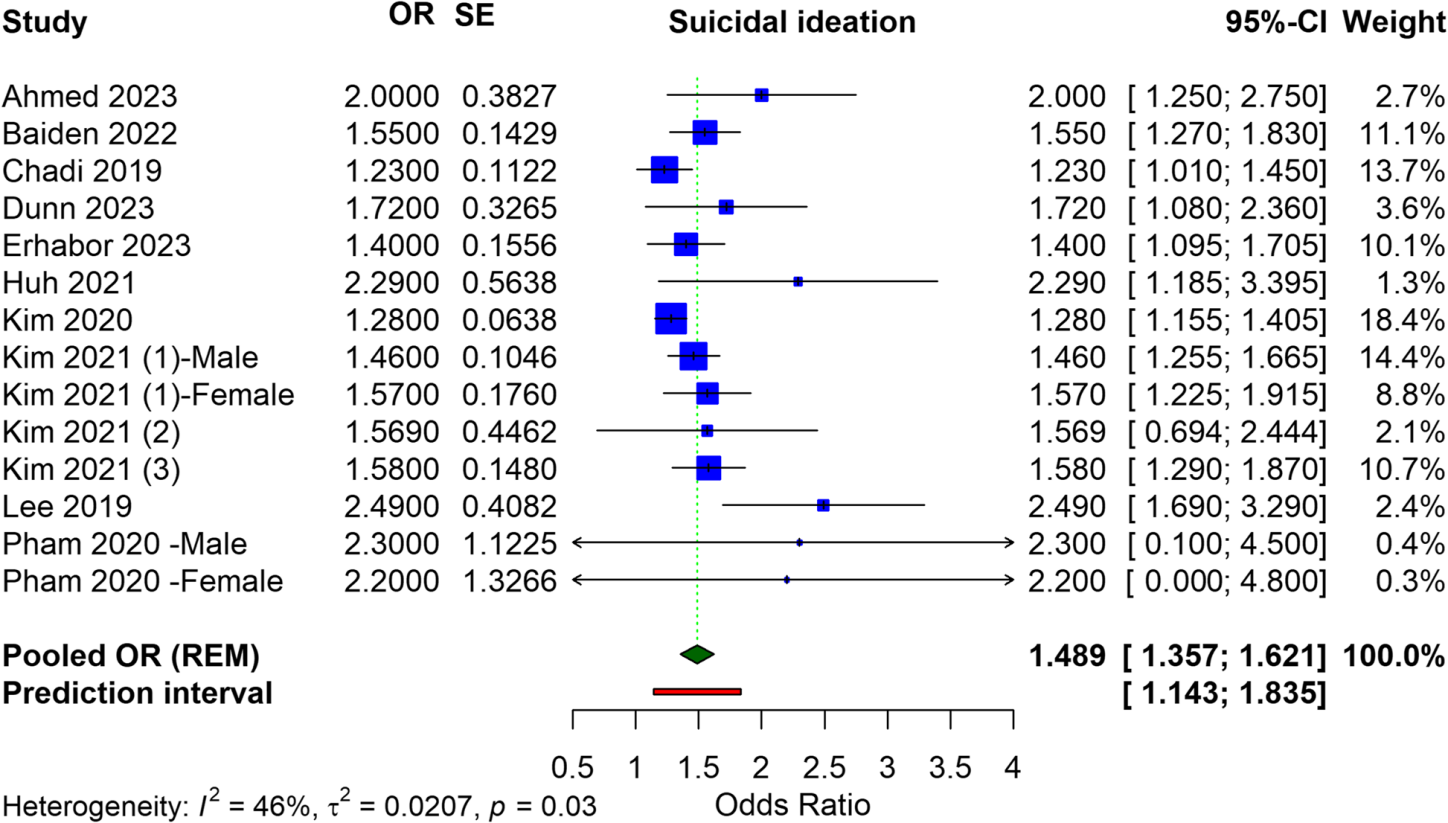
Forest plot illustrating the meta-analysis of association of e-cigarettes and suicidal ideation

The meta-analysis for suicidal ideation associated with electronic cigarette use incorporated data from various studies, yielding pooled ORs. The pooled OR, derived using a REM, stands at 1.489 (95% CI: 1.357 to 1.621), indicating a significant relationship between e-cigarette use and an increased risk of suicidal ideation. This pooled estimate, while accounting for the variability among individual study results, underscores a nearly 50% increase in the odds of suicidal ideation among e-cigarette users versus non-users. The prediction interval (1.143 to 1.835) suggests that the true effect in an individual study is expected to lie within this range 95% of the time. The heterogeneity of the analysis, as shown by an I^2^ value of 46%, suggests moderate variability (Fig. 2). In adolescents or children who use e-cigarettes, the pooled OR for suicide ideation was found to be 1.46 (95% CI: 1.332 to 1.589), with a heterogeneity of I^2^ = 56% (Figure S1).

### Suicidal plan

**Fig. 3:**
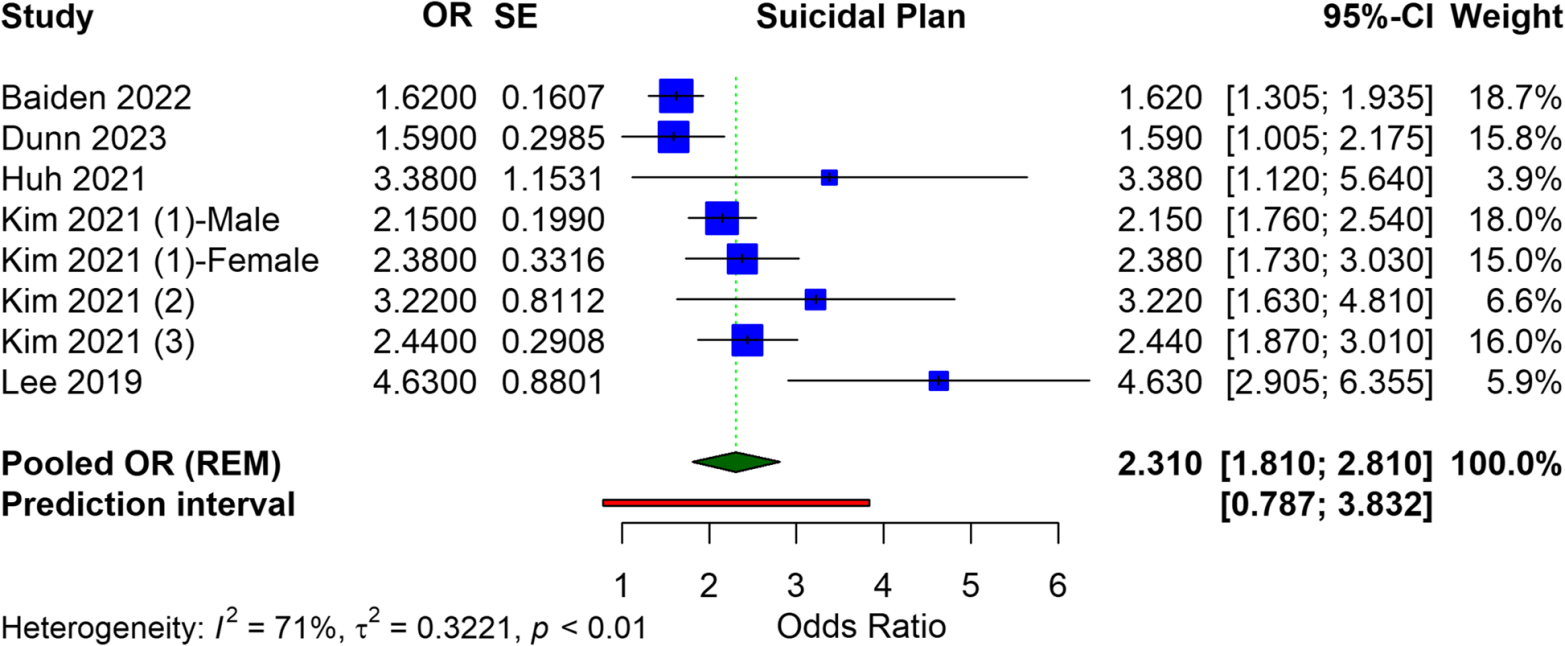
Forest plot illustrating the meta-analysis of association of e-cigarettes and suicidal plan

In the meta-analysis examining the relationship between e-cigarette use and suicidal planning, a pooled analysis of the selected studies was conducted. The OR for suicidal planning is calculated at 2.310 (95% CI: 1.810 to 2.810), suggesting a statistically significant relationship between e-cigarette use and a heightened risk of suicidal planning. The heterogeneity among the studies, indicated by an I^2^ value of 71%, is considered substantial. The prediction interval, from 0.787 to 3.832, also indicates the expected range of true effects in similar future studies, acknowledging the variability observed in the current analysis. This wide range suggests that while the overall trend points towards increased risk, individual studies may report varying degrees of association (Fig. 3). In adolescents or children who use e-cigarettes, the pooled OR for suicide planning was found to be 2.226 (95% CI: 1.739 to 2.712), with a heterogeneity of I^2^ = 73% (Figure S2).

### Suicidal attempt

**Fig. 4:**
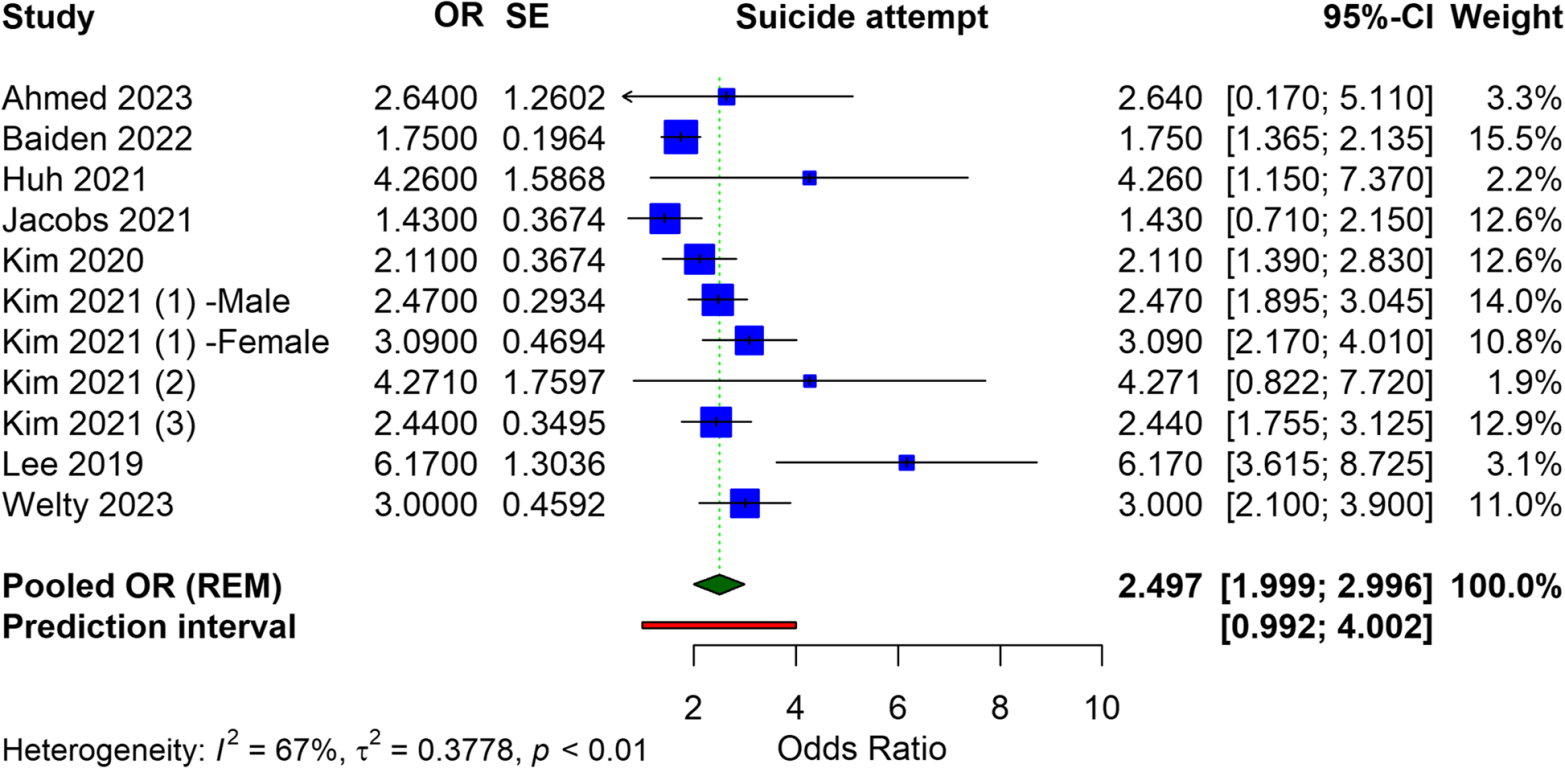
Forest plot illustrating the meta-analysis of association of e-cigarettes and suicide attempt

In the meta-analysis regarding the link between e-cigarette use and suicide attempts, the selected studies encompassed a variety of populations and settings, providing a broad perspective on the issue. The ORs from individual studies point toward a relationship between e-cigarette use and an elevated risk of suicide attempts. The aggregated results yield a pooled OR of 2.497 (95% CI: 1.999 to 3.996), which indicates a significant risk of suicide attempts among e-cigarette users. The heterogeneity of the analysis is high, with an I^2^ value of 67%. Despite this variability, the prediction interval, ranging from 0.992 to 4.002, suggests that future studies are likely to find a true effect within this range, supporting the presence of a linkage between e-cigarette use and suicide attempts (Fig. 4). In adolescents or children who use e-cigarettes, the pooled OR for suicide attempts was found to be 2.462 (95% CI: 1.944 to 2.980), with a heterogeneity of I^2^ = 72% (Figure S3).

### Sensitivity analysis

We performed a sensitivity analysis using the leave-one-out approach, and no significant variation in the results was noted for any of the outcomes. All outcomes remain significantly associated with e-cigarette use in the sensitivity analysis (Figures S4–S6).

### Publication bias

**Fig. 5:**
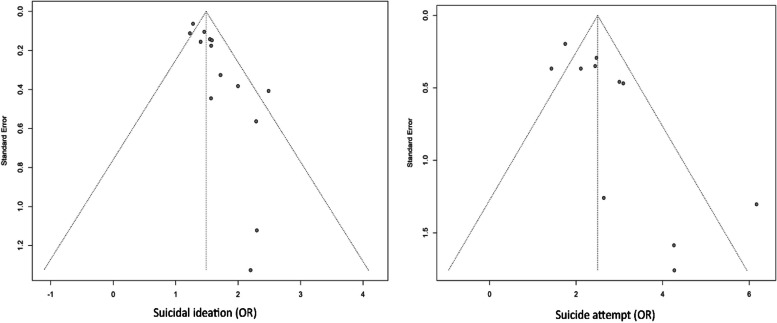
Funnel plot showing publication bias assessment

The assessment for publication bias in our meta-analysis was conducted through visual inspection of funnel plots and the Egger test for suicidal attempts and ideation, as presented in Fig. 5. Due to the limited number of studies available, we did not statistically evaluate publication bias for suicidal planning. Visual inspection of the funnel plots revealed asymmetry. Furthermore, the Egger test results (*p* = 0.0008 for suicidal ideation, *p* = 0.0143 for suicidal attempts) also indicated the presence of publication bias.

We further performed trim and fill analyses (Figures S7 and S8). The OR for suicide ideation was found to be 1.3865 (95% CI: 1.2459 to 1.5271), and for suicide attempts, it was 2.0404 (95% CI: 1.3660 to 2.7148). These results indicate that, even without the effects of small studies, the association between e-cigarette use and suicidal behaviors remains significant.

## Discussion

The findings from our study provide strong evidence of a significant association between e-cigarette use and an increased risk of suicidal behaviors, including ideation, plan and attempts. This is particularly notable given the rising prevalence of e-cigarette use, especially among adolescents and young adults. Our analysis indicates a nearly 50% increase in the odds of suicidal ideation and more than a doubling in the risk of suicide attempts among e-cigarette users. These associations persisted in general population and adolescents, suggesting a potential universal relationship between e-cigarette use and suicidal behaviors rather than one confined to specific contexts.

The potential mechanisms underlying the relationship between e-cigarette use and suicidal behaviors could be multifactorial. Nicotine is a primary component in most e-cigarettes and is known to influence neurotransmitter activity within the brain, potentially altering mood [23]. Additionally, nicotine dependence may contribute to psychological stress, a well-documented risk factor for suicidal behaviors. This is particularly concerning as nicotine can impact neurobiological changes during critical periods of brain development among adolescents, underscoring the potential for e-cigarettes to affect mental health in this vulnerable group.

The findings of this study might also be shaped by cultural and social factors that can influence both e-cigarette usage and suicidal behaviors. Different cultural attitudes towards mental health, smoking, and the acceptance of e-cigarettes can significantly affect how individuals perceive and engage with these products [47, 48]. Social norms, the prevalence of e-cigarette advertising, and the level of awareness about mental health issues vary widely across different regions and cultures, potentially impacting the prevalence of e-cigarette use and its association with suicidal behaviors [49]. Therefore, cultural context and social dynamics variations are essential considerations when interpreting the results and could explain differences in the observed outcomes across different populations.

Prior systematic reviews have explored the impact of e-cigarettes on mental health. For example, Becker et al.'s study highlighted increased mental health issues in young e-cigarette users relative to non-users, particularly in adolescents [17]. Beyond mental health, e-cigarettes have been associated to a range of adverse health outcomes, such as addiction, poisoning, inhalation toxicity, cardiovascular alterations, and diminished lung function as indicated by an umbrella review [50]. Furthermore, the use of e-cigarettes among individuals who have never smoked is linked with an increased likelihood of tobacco smoking initiation, potentially leading to regular smoking habits [51]. A previous scoping review found that suicide attempts were notably more frequent in individuals who used e-cigarettes compared to those who did not [33]. The use of e-cigarettes was linked with depression, suicidal ideation, and suicide attempts [33]. There was a significant increase in suicide attempts among e-cigarette users relative to non-users. These results are comparable to ours.

E-cigarettes, frequently advertised as safer substitutes for conventional cigarettes and as assistance for quitting smoking, are at the centre of ongoing debates. The current evidence on their efficacy and safety is mixed, with no long-term conclusive data to confirm their effectiveness in helping individuals quit tobacco smoking. While some randomized controlled trials (RCTs) have indicated that e-cigarette might assist some smokers in reducing or quitting tobacco use, numerous observational studies have not found significant benefits in quitting tobacco through e-cigarette [52, 53]. Additionally, e-cigarette have associated health risks, such as the potential for nicotine addiction among non-smokers, particularly youth [54, 55].

The regulation of ENDS poses a complex challenge, especially in limiting youth access. The legislative approach to ENDS varies globally, with some countries implementing stringent regulations or outright bans and others having little to no controls. The WHO reports that while 34 countries have banned e-cigarette sales, 88 countries do not restrict age for purchasing these products [56]. Preventing ENDS from becoming a gateway to tobacco smoking for young people necessitates re-evaluation of their accessibility to this demographic. Effective regulation requires ongoing surveillance of ENDS use among both adults and youth, public health initiatives to inform about potential benefits for smokers and risks for non-smokers and young people, and policies mandating the reporting of adverse events.

Our study has some limitations. The cross-sectional nature of the majority of the included studies limits the ability to establish causality. There is also significant variability in the measurement of e-cigarette use and suicidal behaviors across the studies, which could contribute to the heterogeneity of our findings. The lack of longitudinal studies prevents a more thorough examination of the temporal relationship between these variables. Additionally, the majority of the research is concentrated in the USA and Korea, which may limit the generalizability of our findings to other regions. The presence of publication bias is also a concern, as studies with non-significant findings may be underrepresented in the literature. Our analysis might not have fully considered all relevant demographic variables that could influence the relationship between e-cigarette use and suicidal behaviors, such as socioeconomic status or underlying mental health conditions. Finally, the rapid evolution of e-cigarette products and the variability in their nicotine content might not have been fully accounted for, which could affect the applicability of our results to all e-cigarette users.

## Conclusion

Our analysis provided evidence of a relationship between e-cigarette use and an increased risk of suicidal ideation, plan, and attempts. The findings highlight the need for caution among health professionals and policy-makers in promoting e-cigarettes as safe alternatives to smoking. They also point to the necessity for further, high-quality research to explore the interventions aimed at reducing suicidal behaviors among e-cigarette users. Given the significant public health implications, a coordinated effort to address the mental health risks associated with e-cigarette use is warranted.

## Supplementary Information

Supplementary Material 1

## Data Availability

The data is with the authors and available on request to the corresponding author (HAS), Email: habuserhan@hamad.qa.
