# Supplementary material for: Association of electronic cigarette use and suicidal behaviors: a systematic review and meta-analysis
Source: BMC Psychiatry. 2024 Sep 10;24:608. doi: 10.1186/s12888-024-06012-7 (PMC11389297; doi:10.1186/s12888-024-06012-7)
Supplement: Supplementary file 1 — Supplementary Material 1 [file 12888_2024_6012_MOESM1_ESM.docx]

**Supplementary Materials**

**Table S1.** PRISMA Checklist

**Table S2.** Inclusion and Exclusion criteria

**Table S3.** The adjusted search terms as per searched electronic databases

**Table S4.** Modified Newcastle-Ottawa Scale for the quality assessment of studies

**Figure S1**: Association of e-cigarettes and suicide ideation among adolescents/children

**Figure S2**: Association of e-cigarettes and suicide plan among adolescents/children

**Figure S3**: Association of e-cigarettes and suicide attempts among adolescents/children

**Figure S4:** Sensitivity analysis of suicide ideation

**Figure S5:** Sensitivity analysis of suicide plan

**Figure S6:** Sensitivity analysis of suicide attempts

**Figure S7:** Trim and fill funnel plot for suicide ideation

**Figure S8:** Trim and fill funnel plot for suicide attempt

## **Table S1.** PRISMA Checklist

| **Section and Topic** | **Item #** | **Checklist item (Prevalence of kidney diseases among the dengue patients: A systematic review and meta-analysis)** | **Location where item is reported** |
| --- | --- | --- | --- |
| **TITLE** | | |  |
| Title | 1 | Identify the report as a systematic review. | 1 |
| **ABSTRACT** | | |  |
| Abstract | 2 | See the PRISMA 2020 for Abstracts checklist. (made as per the Journal guidelines) | 2 |
| **INTRODUCTION** | | |  |
| Rationale | 3 | Describe the rationale for the review in the context of existing knowledge. | 3 |
| Objectives | 4 | Provide an explicit statement of the objective(s) or question(s) the review addresses. | 3 |
| **METHODS** | | |  |
| Eligibility criteria | 5 | Specify the inclusion and exclusion criteria for the review and how studies were grouped for the syntheses. | 4 |
| Information sources | 6 | Specify all databases, registers, websites, organisations, reference lists and other sources searched or consulted to identify studies. Specify the date when each source was last searched or consulted. | 4, Table S3 |
| Search strategy | 7 | Present the full search strategies for all databases, registers and websites, including any filters and limits used. | Table S3 |
| Selection process | 8 | Specify the methods used to decide whether a study met the inclusion criteria of the review, including how many reviewers screened each record and each report retrieved, whether they worked independently, and if applicable, details of automation tools used in the process. | 4 |
| Data collection process | 9 | Specify the methods used to collect data from reports, including how many reviewers collected data from each report, whether they worked independently, any processes for obtaining or confirming data from study investigators, and if applicable, details of automation tools used in the process. | 4 |
| Data items | 10a | List and define all outcomes for which data were sought. Specify whether all results that were compatible with each outcome domain in each study were sought (e.g., for all measures, time points, analyses), and if not, the methods used to decide which results to collect. | 3 |
|  | 10b | List and define all other variables for which data were sought (e.g., participant and intervention characteristics, funding sources). Describe any assumptions made about any missing or unclear information. | 4, Table S4 |
| Study risk of bias assessment | 11 | Specify the methods used to assess risk of bias in the included studies, including details of the tool(s) used, how many reviewers assessed each study and whether they worked independently, and if applicable, details of automation tools used in the process. | Table S4 |
| Effect measures | 12 | Specify for each outcome the effect measure(s) (e.g. risk ratio, mean difference) used in the synthesis or presentation of results. | 5 |
| Synthesis methods | 13a | Describe the processes used to decide which studies were eligible for each synthesis (e.g. tabulating the study intervention characteristics and comparing against the planned groups for each synthesis (item #5)). | 4,5 |
|  | 13b | Describe any methods required to prepare the data for presentation or synthesis, such as handling of missing summary statistics, or data conversions. | NA |
|  | 13c | Describe any methods used to tabulate or visually display results of individual studies and syntheses. |  |
|  | 13d | Describe any methods used to synthesize results and provide a rationale for the choice(s). If meta-analysis was performed, describe the model(s), method(s) to identify the presence and extent of statistical heterogeneity, and software package(s) used. | 4.5 |
|  | 13e | Describe any methods used to explore possible causes of heterogeneity among study results (e.g. subgroup analysis, meta-regression). | 5 |
|  | 13f | Describe any sensitivity analyses conducted to assess robustness of the synthesized results. | NA |
| Reporting bias assessment | 14 | Describe any methods used to assess risk of bias due to missing results in a synthesis (arising from reporting biases). | 5 |
| Certainty assessment | 15 | Describe any methods used to assess certainty (or confidence) in the body of evidence for an outcome. | NA |
| **RESULTS** | | |  |
| Study selection | 16a | Describe the results of the search and selection process, from the number of records identified in the search to the number of studies included in the review, ideally using a flow diagram. | Table S2 |
|  | 16b | Cite studies that might appear to meet the inclusion criteria, but which were excluded, and explain why they were excluded. | NA |
| Study characteristics | 17 | Cite each included study and present its characteristics. | 4,5 Table 1 |
| Risk of bias in studies | 18 | Present assessments of risk of bias for each included study. | Table S4 |
| Results of individual studies | 19 | For all outcomes, present, for each study: (a) summary statistics for each group (where appropriate) and (b) an effect estimate and its precision (e.g. confidence/credible interval), ideally using structured tables or plots. | Table 1, Figure 2 |
| Results of syntheses | 20a | For each synthesis, briefly summarise the characteristics and risk of bias among contributing studies. | 4 |
|  | 20b | Present results of all statistical syntheses conducted. If meta-analysis was done, present for each the summary estimate and its precision (e.g. confidence/credible interval) and measures of statistical heterogeneity. If comparing groups, describe the direction of the effect. | 5,4 Figure 2 |
|  | 20c | Present results of all investigations of possible causes of heterogeneity among study results. | 5, Figure S1-S6 |
|  | 20d | Present results of all sensitivity analyses conducted to assess the robustness of the synthesized results. | Figure S4-S6 |
| Reporting biases | 21 | Present assessments of risk of bias due to missing results (arising from reporting biases) for each synthesis assessed. | Figure 5 |
| Certainty of evidence | 22 | Present assessments of certainty (or confidence) in the body of evidence for each outcome assessed. | NA |
| **DISCUSSION** | | |  |
| Discussion | 23a | Provide a general interpretation of the results in the context of other evidence. | 5,6, 7 |
|  | 23b | Discuss any limitations of the evidence included in the review. | 7 |
|  | 23c | Discuss any limitations of the review processes used. | 7 |
|  | 23d | Discuss implications of the results for practice, policy, and future research. | 7 |
| **OTHER INFORMATION** | | |  |
| Registration and protocol | 24a | Provide registration information for the review, including register name and registration number, or state that the review was not registered. | 3 |
|  | 24b | Indicate where the review protocol can be accessed, or state that a protocol was not prepared. | 3 |
|  | 24c | Describe and explain any amendments to information provided at registration or in the protocol. | NA |
| Support | 25 | Describe sources of financial or non-financial support for the review, and the role of the funders or sponsors in the review. | 8 |
| Competing interests | 26 | Declare any competing interests of review authors. | 7 |
| Availability of data, code and other materials | 27 | Report which of the following are publicly available and where they can be found: template data collection forms; data extracted from included studies; data used for all analyses; analytic code; any other materials used in the review. | Supplementary Materials |

**Table S2. The adjusted search terms as per searched electronic databases [as of 10.03.2024]**

| **Database** | **Search query** | **Result** |
| --- | --- | --- |
| **PubMed** | ("e cig*"[All Fields] OR "vaper*"[All Fields] OR ("vaping"[MeSH Terms] OR "vaping"[All Fields] OR "vape"[All Fields] OR "electronic nicotine delivery systems"[MeSH Terms] OR ("electronic"[All Fields] AND "nicotine"[All Fields] AND "delivery"[All Fields] AND "systems"[All Fields]) OR "electronic nicotine delivery systems"[All Fields]) OR "electronic cig*"[All Fields] OR "electronic nicot*"[All Fields]) AND ("Suicide"[All Fields] OR "self harm*"[All Fields] OR ("suicid"[All Fields] OR "suicidal ideation"[MeSH Terms] OR ("suicidal"[All Fields] AND "ideation"[All Fields]) OR "suicidal ideation"[All Fields] OR "suicidality"[All Fields] OR "suicidal"[All Fields] OR "suicidally"[All Fields] OR "suicidals"[All Fields] OR "Suicide"[MeSH Terms] OR "Suicide"[All Fields] OR "suicides"[All Fields] OR "suicide s"[All Fields] OR "suicided"[All Fields] OR "suiciders"[All Fields])) | **80** |
| **Web of Science** | (“e-cig*” OR “vaper*” OR vape OR “electronic cig*” OR “electronic nicot*”) AND (“Suicide” OR “self harm*” OR suicidal) | **58** |
| **EMBASE** | (“e-cig*” OR “vaper*” OR vape OR “electronic cig*” OR “electronic nicot*”) AND (“Suicide” OR “self harm*” OR suicidal) | **150** |

**Table S3.** Inclusion and Exclusion criteria

**Research Question:** Is Electronic cigarette use associated with suicidal behaviours?

| **Inclusion** | | **Exclusion** |
| --- | --- | --- |
| **Participants** | General population | None |
| **Exposure** | E-cigarettes, Vaping, ENDS | Tobacco smoking |
| **Outcome** | Suicidal ideation, suicidal plan, suicidal attempts | Non-suicidal self-harms |
| **Study Designs** | Observational studies, cross-sectional studies, longitudinal studies, retrospective studies, prospective studies, case-control studies, | Qualitative, policy, opinion, case studies, case reports, reviews, and animal studies. |
|  | Geography-Global level  Date of Search- Publish till 010^th^ March 2024 | None |
|  | Published articles |  |

**Table S4.** Modified Newcastle-Ottawa Scale tool for the quality assessment of studies

| **Sl.no** | **Study** | **Representativeness** | **Sample size 500** | **Definition** | **Ascertainment of suicidal behavious** | **Total** |
| --- | --- | --- | --- | --- | --- | --- |
|  | Ahmed 2023 (1) | 1 | 1 | 1 | 1 | 4 |
|  | Baiden 2022 (2) | 2 | 1 | 2 | 1 | 6 |
|  | Chadi 2019 (3) | 2 | 1 | 1 | 1 | 5 |
|  | Dunn 2023 (4) | 1 | 1 | 2 | 1 | 5 |
|  | Erhabor 2023 (5) | 1 | 1 | 1 | 1 | 4 |
|  | Huh 2021 (6) | 2 | 0 | 2 | 1 | 5 |
|  | Jacobs 2021 (7) | 1 | 1 | 1 | 1 | 4 |
|  | Kim 2020 (8) | 2 | 1 | 1 | 1 | 5 |
|  | Kim 2021 (9) | 2 | 1 | 2 | 1 | 6 |
|  | Kim 2021 (10) | 2 | 1 | 1 | 1 | 5 |
|  | Kim 2021 (11) | 2 | 0 | 1 | 1 | 4 |
|  | Lee 2019 (12) | 1 | 0 | 2 | 1 | 4 |
|  | Pham 2020 (13) | 2 | 0 | 1 | 1 | 4 |
|  | Welty 2023 (14) | 2 | 0 | 2 | 1 | 5 |

**
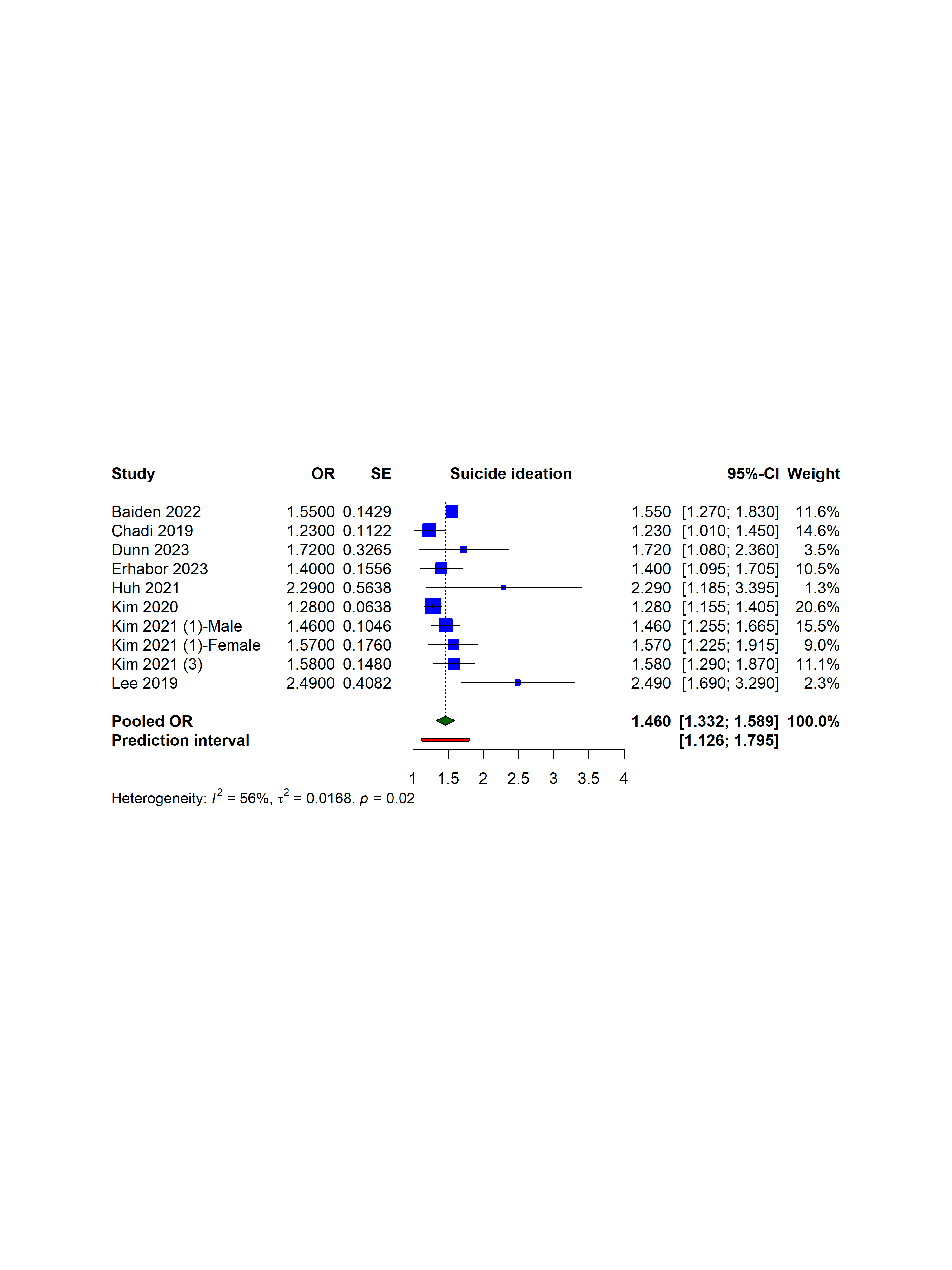
**

**Figure S1**: Association of e-cigarettes and suicide ideation among adolescents/children


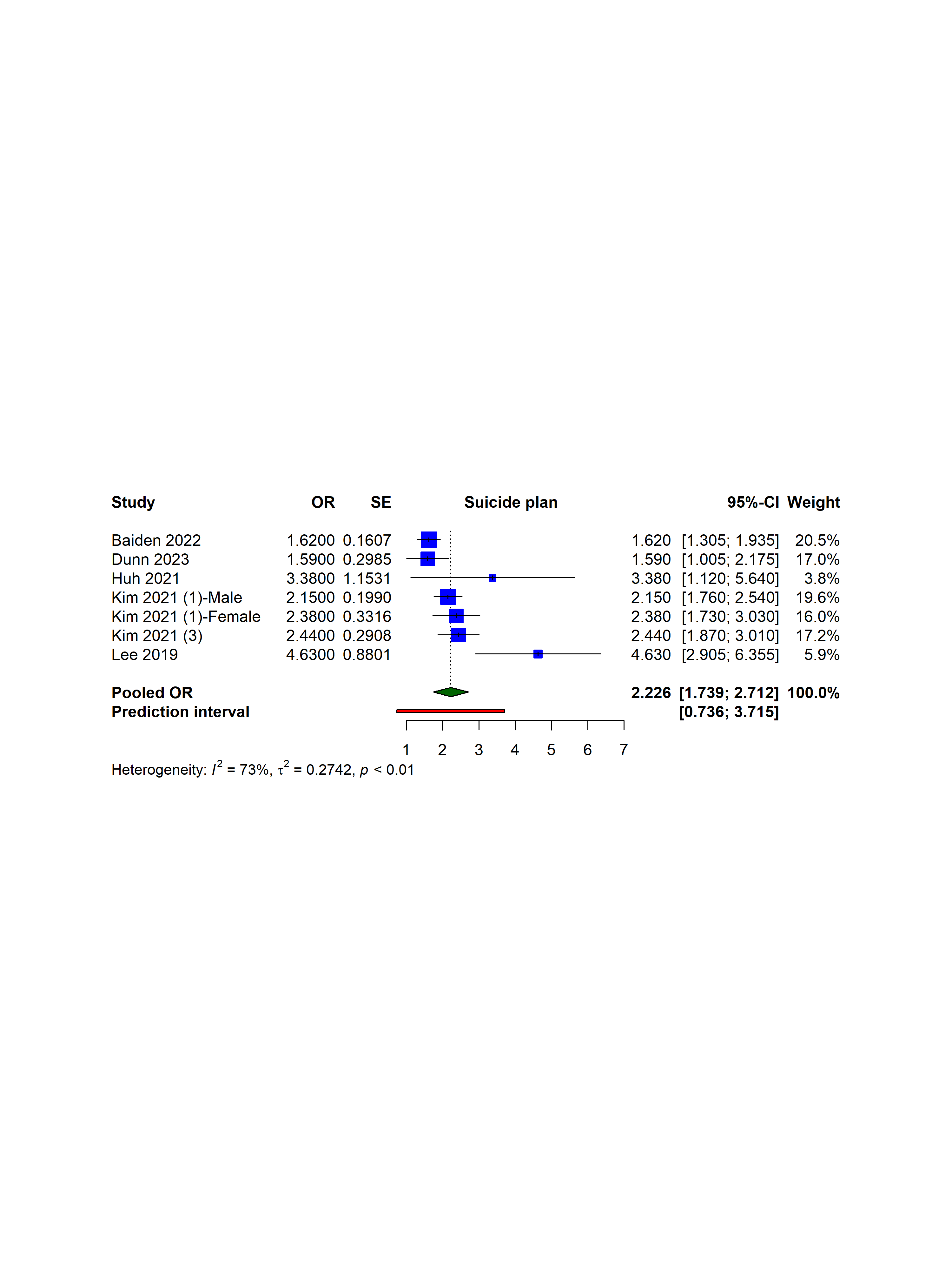


**Figure S2**: Association of e-cigarettes and suicide plan among adolescents/children


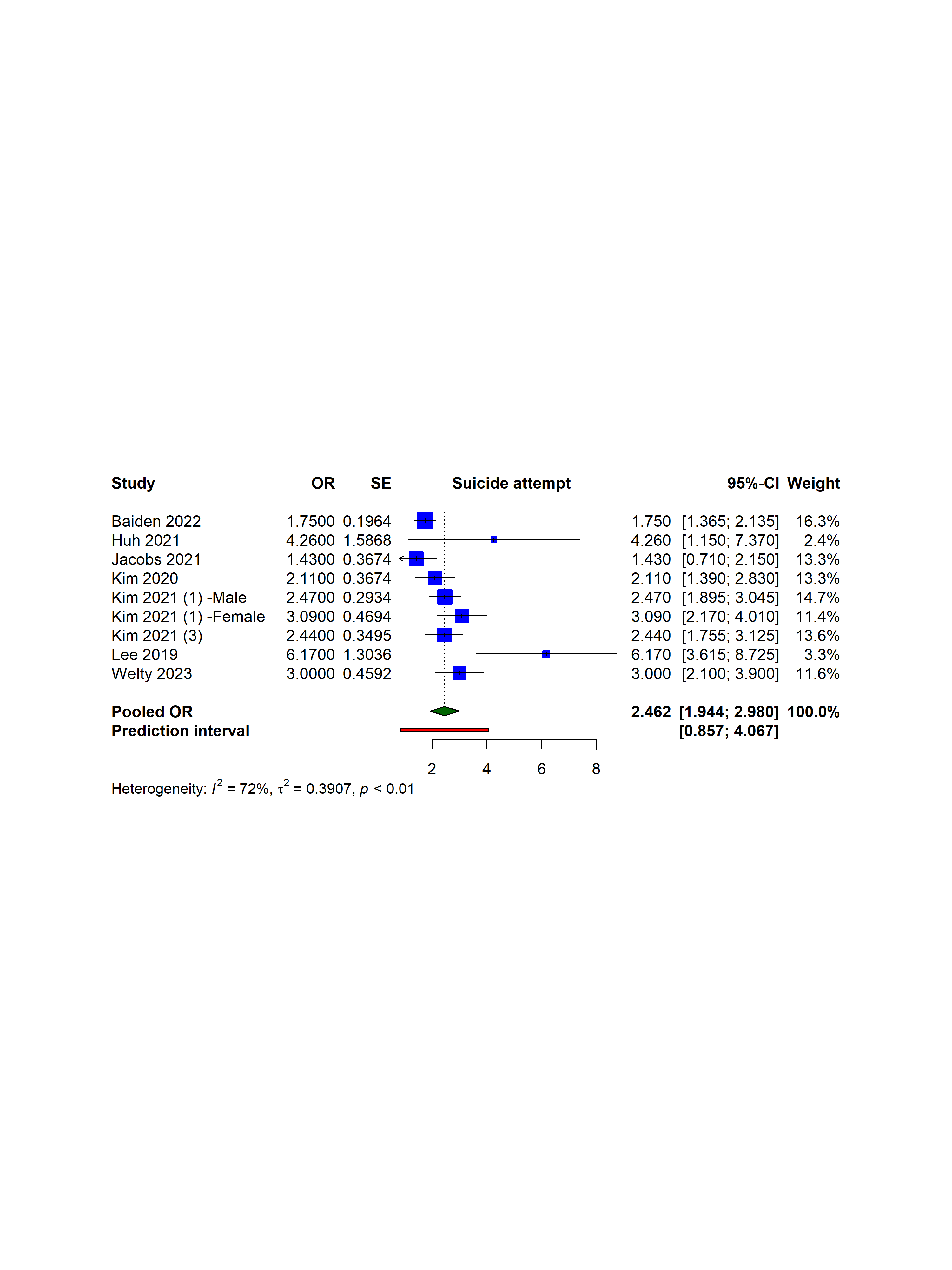


**Figure S3**: Association of e-cigarettes and suicide attempts among adolescents/children


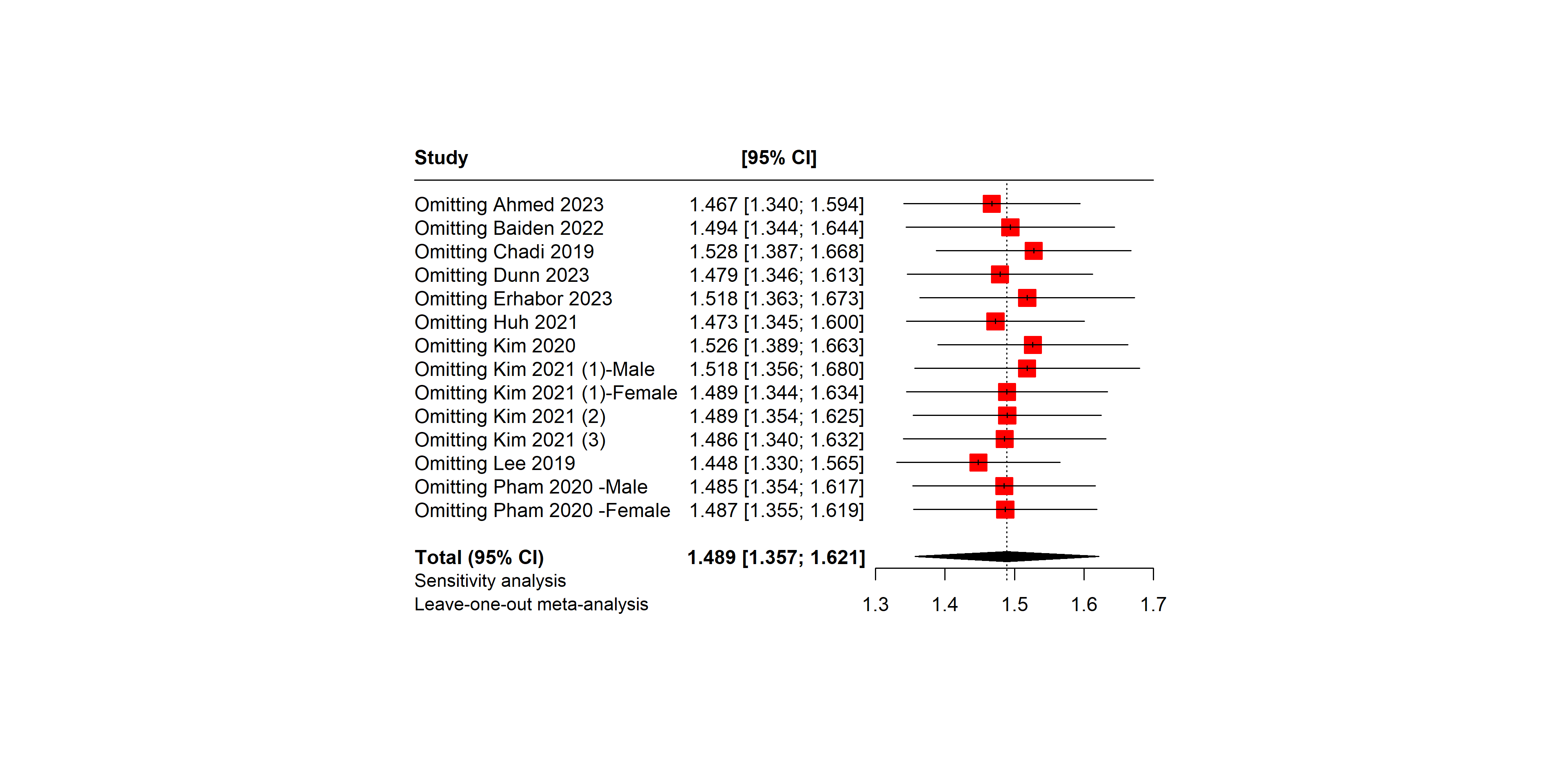


**Figure S4:** Sensitivity analysis of suicide ideation


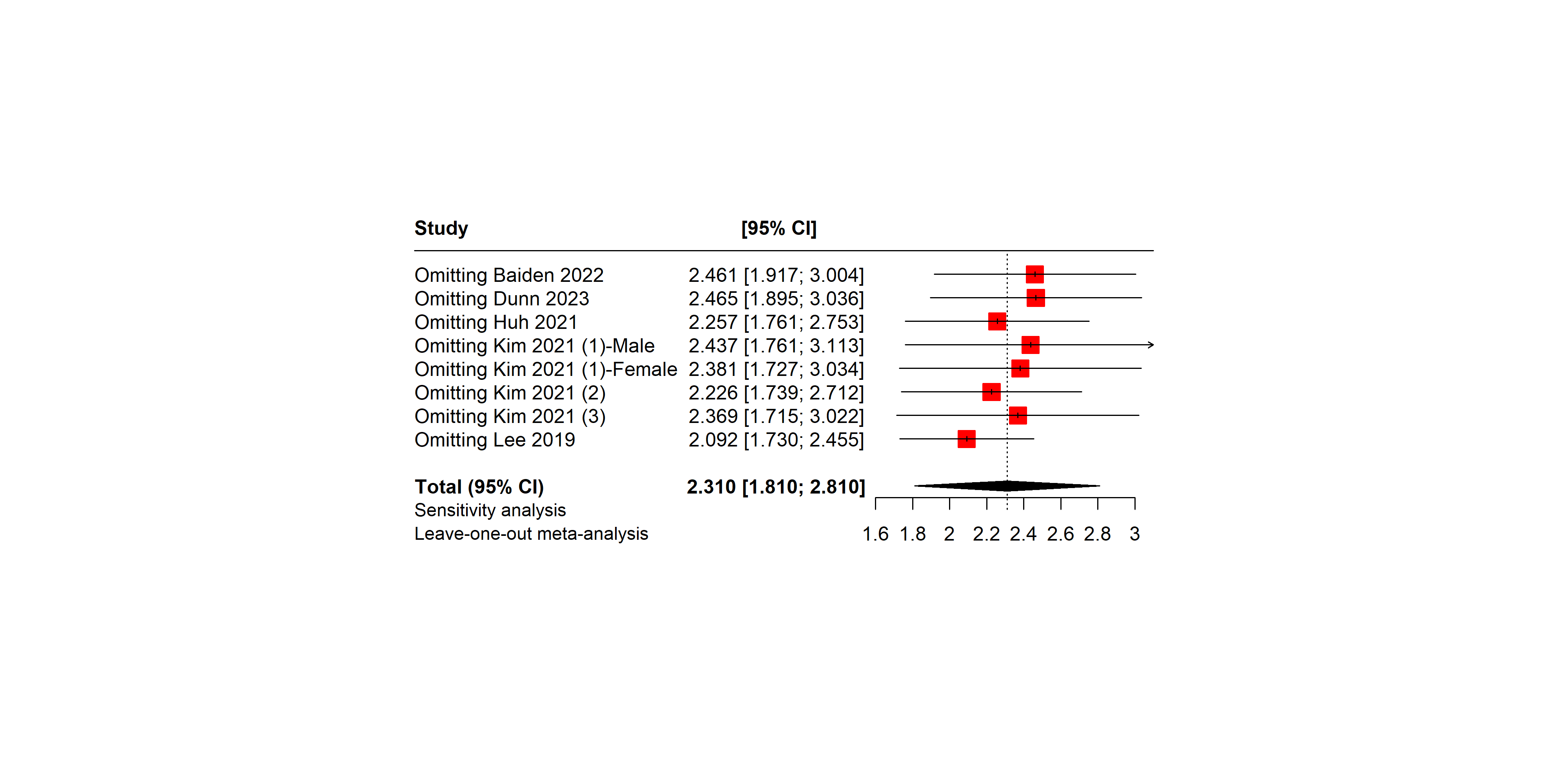


**Figure S5:** Sensitivity analysis of suicide plan


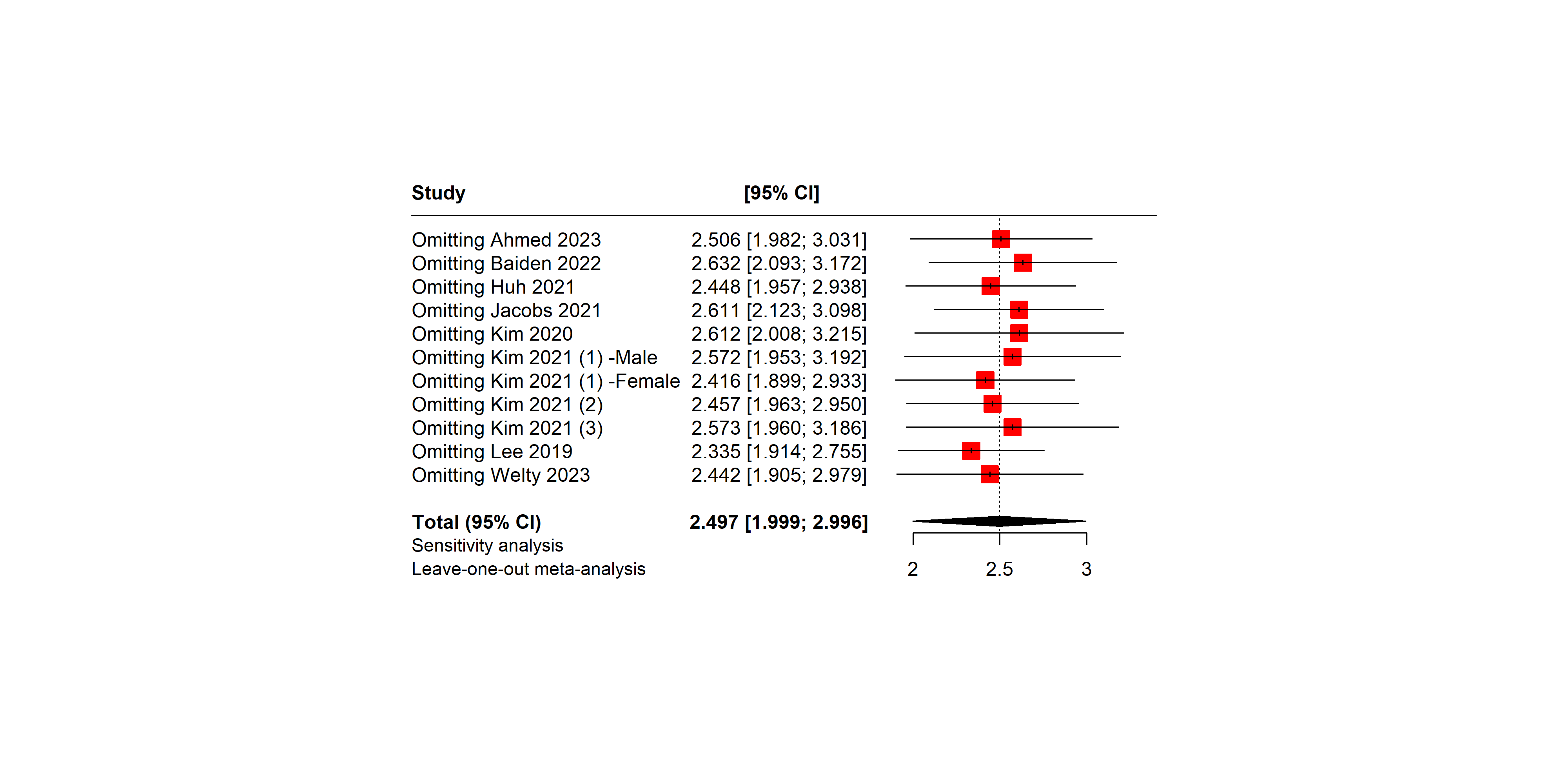


**Figure S6:** Sensitivity analysis of suicide attempts


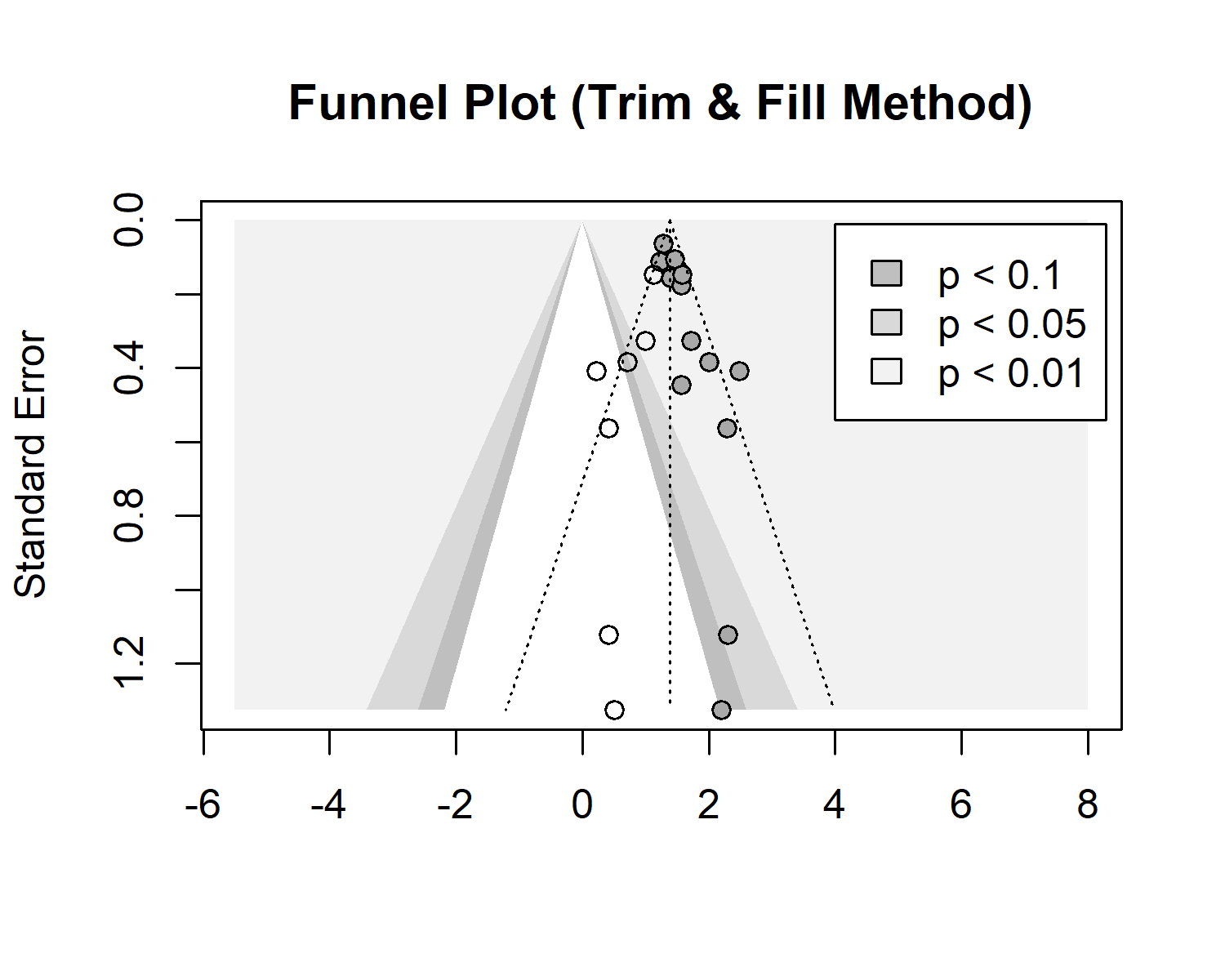


**Figure S7:** Trim and fill funnel plot for suicide ideation


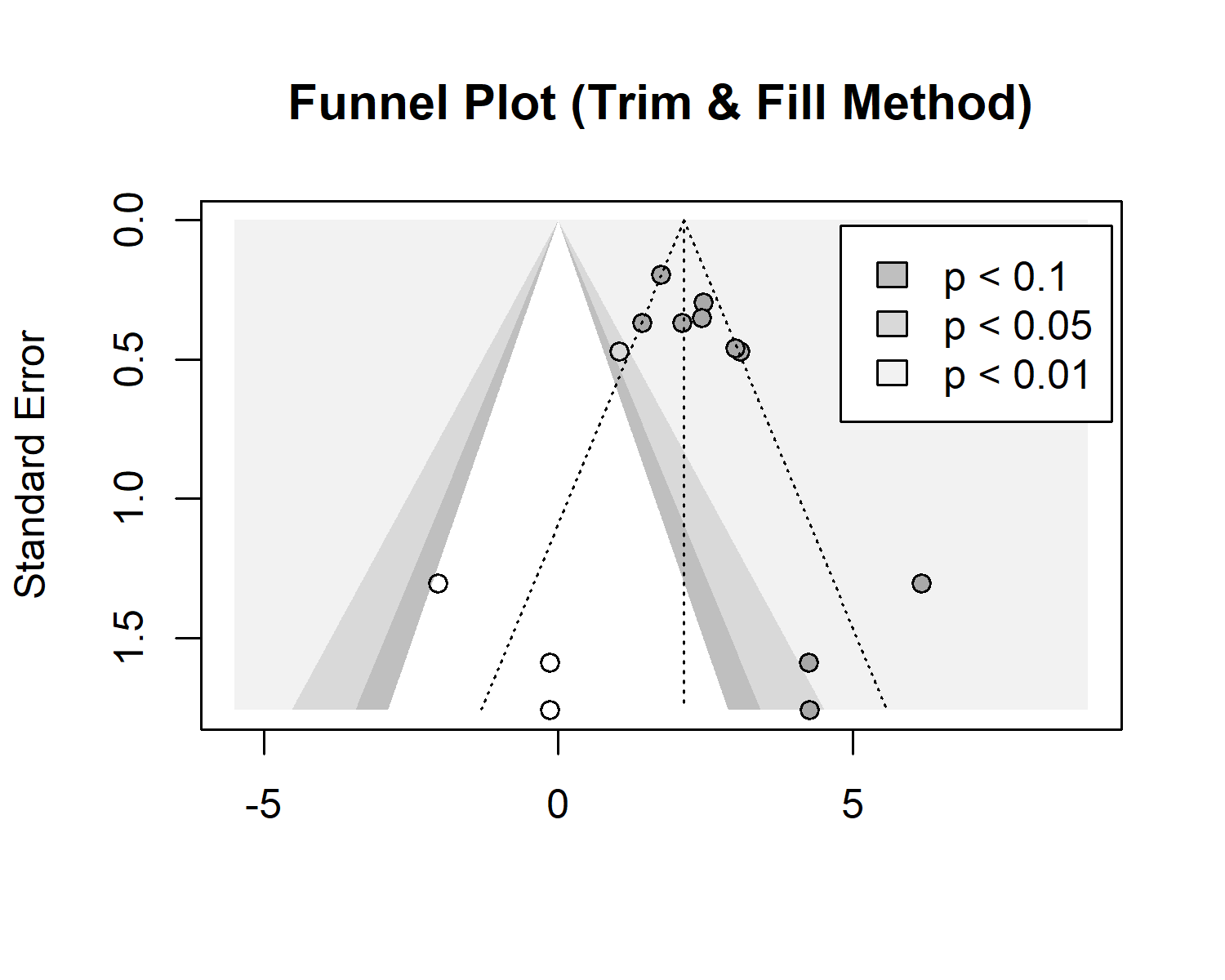


**Figure S8:** Trim and fill funnel plot for suicide attempt

**Bibliography**

1. Ahmed AE, Yim MH, Dawood J, Olsen CH, Waters AJ, Singer DE, Mancuso JD. Suicidal behaviors among active-duty US service members: data from the 2018 health-related behaviors survey. Psychology research and behavior management. 2023:4599-615.

2. Baiden P, Szlyk HS, Cavazos-Rehg P, Onyeaka HK, Peoples JE, Kasson E. Use of electronic vaping products and mental health among adolescent high school students in the United States: The moderating effect of sex. Journal of psychiatric research. 2022;147:24-33.

3. Chadi N, Li G, Cerda N, Weitzman ER. Depressive symptoms and suicidality in adolescents using e-cigarettes and marijuana: a secondary data analysis from the youth risk behavior survey. Journal of addiction medicine. 2019;13(5):362-5.

4. Dunn MS, Yannessa JF. Suicide ideation and behavior and ATOD use among bisexual high school students. Journal of Substance Use. 2023:1-5.

5. Erhabor J, Boakye E, Osuji N, Obisesan O, Osei AD, Mirbolouk H, et al. Psychosocial stressors and current e-cigarette use in the youth risk behavior survey. BMC public health. 2023;23(1):1080.

6. Huh Y, Cho H-J. Associations between the type of tobacco products and suicidal behaviors: a nationwide population-based study among Korean adolescents. International journal of environmental research and public health. 2021;18(2):367.

7. Jacobs W, Idoko E, Montgomery L, Smith ML, Merianos AL. Concurrent E-cigarette and marijuana use and health-risk behaviors among US high school students. Preventive medicine. 2021;145:106429.

8. Kim CW, Jeong SC, Kim JY, Lee JS, Lee JH, Jo SH, Kim SH. Associated factors for depression, suicidal ideation and suicide attempt among asthmatic adolescents with experience of electronic cigarette use. Tobacco induced diseases. 2020;18.

9. Kim SH, Jeong SH, Park E-C, Jang S-I. Association of cigarette type initially smoked with suicidal behaviors among adolescents in Korea from 2015 to 2018. JAMA network open. 2021;4(4):e218803-e.

10. Kim JY, Kang HS, Jung J-W, Jung SY, Park HJ, Park JS, et al. Nicotine dependence and stress susceptibility in E-cigarette smokers: The Korea national health and nutrition examination survey 2013–2017. Tuberculosis and Respiratory Diseases. 2021;84(2):159.

11. Kim JS, Kim K. Electronic cigarette use and suicidal behaviors among adolescents. Journal of Public Health. 2021;43(2):274-80.

12. Lee Y, Lee K-S. Association of depression and suicidality with electronic and conventional cigarette use in South Korean adolescents. Substance use & misuse. 2019;54(6):934-43.

13. Pham T, Williams JV, Bhattarai A, Dores AK, Isherwood LJ, Patten SB. Electronic cigarette use and mental health: a Canadian population-based study. Journal of Affective Disorders. 2020;260:646-52.

14. Welty CW, Gerald LB, Nair US, Haynes PL. Vaping and sleep as predictors of adolescent suicidality. American journal of health promotion. 2023;37(1):39-46.
